# 

**Published:** 1887-11-26

**Authors:** 


### SPECIAL NOTICE TO SUBSCRIBERS AND THE TRADE.

NEXT week's issue of THE HOSPITAL will consist of a Double Number, and will contain a supplement, entitled,

"NOT PREACHING NOR PUBLICITY, BUT PRACTICE."

This Supplement will deal exhaustively with the present needs and condition of the unemployed and casual population of London, and will contain a practical solution of the social problems they present.

Orders for this Number should be sent to the publishers without delay.

### NOTICE TO ADVERTISERS.

THE scale of charges for small prepaid Advertisements—Situations Wanted, Situations Vacant, &c , &c., is as follows :—

| | s. | d. |
|---|---|---|
| One insertion of three lines | 1 | 0 |
| Three " " " | 2 | 6 |
| Per line afterwards | 0 | 6 |

*A line averages ten words.*

All copy for Advertisements must be sent to A. P. WATT, 2, PATERNOSTER SQUARE, E.C., by first post on the Tuesday *preceding* publication.

### NOTICE TO CORRESPONDENTS.

All correspondence must be written on one side of the paper only.

We cannot undertake to return MSS. not used.

**Communications,** whether intended for insertion or for private information, must be authenticated by the names and addresses of the writers, not necessarily for publication.

**Local papers** containing reports or news paragraphs **should be marked.**

It is especially requested that early intelligence of **local events of interest,** or which it may be thought desirable to bring under notice, may be sent direct to the Editor.

**All communications respecting editorial matters** should be addressed to the Editor, Norfolk House, Norfolk Street, Strand, London, W.C.

### Amusements with Profit for all Ages.

**Rules.**—All answers must be addressed to the Prize Editor, The Hospital, Norfolk House, Norfolk Street, Strand, W.C., not later than the *first* post on Thursday next. The full name and address must in all cases be given, but a *nom de plume* may be added for publication. Only one side of the paper must be written on.

No one will be permitted to win the Monthly or Quarterly Prizes twice in any one year, the annual prizes being offered especially to prevent this.

### FOR MEN AND WOMEN.

### To our Competitors.

The alterations made in this column have caused some perturbation and excitement amongst those who competed previously. Now that the acrostics have been discontinued, numerous readers discover their liking for this kind of amusement. The changes have, however, infused new life into the competitions, and the competitors now number nearly thirty, where they only numbered six. Here is proof that the change was desirable, but as we are willing to meet the views of all as far as possible, we shall set in future an acrostic to be solved, or give marks for the best original acrostic or some other puzzle sent in, in addition to or in lieu of the word competition, at the competitor's option. Marks will be given according to the work done, and those who obtain the highest marks will secure the prizes. It has been found impossible to arrange to give the yearly prize as at first contemplated in the former competitions. This decision is rendered necessary, to our great regret, owing to the number of competitors having fallen practically to zero after nine months' trial, which compelled us to rearrange the competitions in this column, or to give them up altogether.

### I.—The Quilter Prizes. Six Guineas.

FOR BEST SPECIMENS OF WORK.

Three prizes of three guineas, two guineas, and one guinea, will be given to the competitor who sends in the first, second, and third best specimens of work of any kind, suitable for presentation to the inmates of the Hospitals, addressed to the Editor, The Lodge, Porchester Square, W., on or before the 31st of December next. This work may take the form of a toy, a doll, or a scrap book for the children; an article of clothing, a painting or watercolour, an illuminated card or any other gift, for men or women, or for to aid decoration. It is proposed to arrange for the distribution of the articles sent in for this competition amongst the patients in the hospitals. *Esor* has sent in a scrap-book for competition.

### II.—Word and Literary Competition.

Commenced November 5th, 1887; ends January 28th, 1888.

Three prizes of one guinea, 15*s.* and 10*s.* 6*d.* will be given each quarter to the three competitors who obtain the highest number of marks: (1) by making the largest number of words derived from the word or phrase set for anatomical dissection; or (2) for the best answers to (6); or (3) by (1) and (2) combined.

(A) THE WORD COMPETITION.

We shall give the newspapers for disection during the first quarter, that for this week being "Standard."

Observe.—Proper names, abbreviations, foreign words, words of less than four letters and repetitions are barred. Nuttall's dictionary only to be used.

### Results of Second Week.

*Accepted Scores*—Reldas (3,079), E. E. (3,055), Ellice (2,668), K. M. (1,607), Mater (1,022), Aralc (949), Tim (536), Toby (158).

I. Double Acrostic.

Dying, yet still I live,
New tints to nature give;
Change and decay are mine
Mine by decree divine.
Finals my primals hate,
Primals are final's fate.

Creation of a poet's brain,
A fairy in a mortal's train;
Spell-bound by witch, by mortal freed,
Supplies his master's utmost need.

Once was I soulless, now my love
A soul bestows; and yet I move
In fear, lest any angry mood
Should plunge me soulless in the flood.

Queens, Popes, and ladies all,
In pageant, state, or ball,
Their heads adorn with me,
And more complacently.
Two neighbour letters, once of yore
Used equally by learned lore.

When horrors gloom and deathly doom
Darken the stage of tragic page,
When lightnings flash and thunders crash.
When mortal's fear and ghosts appear
Thy hand is seen, majestic queen,
An artist's sign of grace divine.

(B) THE LITERARY COMPETITION.

Some people hate puzzles of all kinds, including word competitions, but they are fond of correspondence, and many sisters and mothers are continually receiving and sending letters to and from members of the family who are abroad in India or the Colonies. The constant coming and going between England and other portions of the British Empire make it a matter of interest and importance to know something about the life, the climate, the amusements, the adventures, the clothing, and the surroundings of those who seek a livelihood beyond the seas. We shall therefore try to find space for the publication of selected letters from abroad, which may be sent by any of our readers who have friends and relations in foreign lands. We shall give marks for these letters as well as for the word competitions which will count equally for these prizes, so that anyone may enter for both or either, the prizes to be given to those who get the highest number of marks. The first letter from across the seas will be found on page 93 of The Hospital for Nov. 5th last. It is a record of adventure in Australia, and gives a good idea of one class of letter suitable for this column. *Aralc* sends a letter from Syria, *Gretta* another from India.

### THE CHILDREN'S CORNER.

PRIZES.

FOR MOST CORRECT ANSWERS TO PUZZLES.

This Commenced October 1st, 1887.

One Pound a Month.

FOR BEST LITTLE LETTERS.

Ten Shillings per Quarter.

A PRIZE OF THREE GUINEAS

to the Competitor who shall have sent in the largest number of correct or best answers during the twelve months.

### Puzzles.—Fourth Week.

Prizes.

No. 13. Geographical Acrostic.—1. A fortified town in France noted for its broadcloths. 2. A large river in Siberia. 3. A river in Suffolk. 4. A district of British India. 5. An island in the Ægean Sea. Initials will give the name of a town in France, and the finals tell you for what it is noted.

No. 14. Draw the following figure without passing over any line twice and without lifting the pen:—

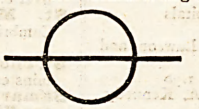

Answers must show by means of an arrow the direction followed by the pen.

No. 15. Biographical Charade.

Traverse the world from Pole to Pole,
You'll find my *first* esteemed by all;
My *second* is an occupation
That's useful found in every nation;
Connect the two, and you'll acquire
An author whom we much admire.

No. 16. Enigma. If my *first* were always my *second*, the whole world would be greatly benefitted by it.

### Letters.

The letter subject for next week will be "St. Paul's Cathedral."

*Three marks each*—The Eda, Gem, Skye, Ossian.
*Two marks each*—Betty Burke, Poodle, Fern.

### Results of Second Week Puzzles.

No. 5. Soups.—Chestnut. Artichoke.
Fish.—Skate. Pike.
Entrees.—Larks and Quails.
Poultry.—Goose.
Sauce.—Apple.
Sweets.—Trifle. Greengage tart.

No. 6. Because it is used to partridges (part ridges).

No. 7. The house was seventy-two years old. Thus—sixty years ago it was twelve years old. The square of twelve is 144, which, divided by two, give seventy-two.

No. 8.

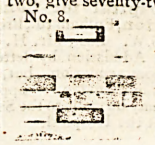

W orcester.

A cre.

T rafalgar.

E vesham.

R oses,

L eipsic.

O tterbourne.

O udenarde.

*All right*—A. C. K., Pepita, G. B. P., Tim, M. S. W. *Three right*—Poodle, Scrooge, Alsie, Paddy, Jumps, The Eda. *Two right*—Skye, Ossian, Mount Edgecumbe, Gem. *One right*—Spider, The Young Canadian, Joe, Judy, Fern, W. R. Mills, Betty Burke, A. G. S. McCulloch.

### THE THEATRES.

Mr. Hawtrey's tenure of the Globe terminates in a week, but he transfers his present piece, *The Arabian Nights*, to the Comedy Theatre, where it bids fair to rival the popularity of the *Private Secretary*, and to exceed that of its predecessor, *The Doctor*. From the rise of the curtain to its fall at the end of the third act, the house resounded with laughter, and on several occasions was fairly convulsed. Mr. Sidney Grundy, the author, has founded his comedy on a German play by Von Moser, but the dialogue is evidently his own, and written in his best style. Some of the repartee is extremely smart, and the complications arising in the play are excellently worked up.

The story is very slight, and depends mainly on the excessive zeal with which Mrs. Gillibrand, the mother-in-law, looks after Arthur Hummingtop during the temporary absence of his wife, and on the antics of her own son, Joshua Gillibrand, and the gutta-percha girl, Rose Columbier, on whom he is "mashed." To wile away the tedium, Arthur Hummingtop studies the "Arabian Nights," is tempted to imitate the wanderings of Haroun-al-Raschid. Chance throws him across Rosa Colombier, with whom he is lost in a fog, but he succeeds at last in helping her to find her destination, the Aquarium. Rosa finds him out next day, and is deaf to all his entreaties to go away. Driven to desperation, Hummingtop introduces her to his mother-in-law as his expected niece from America, and she is installed in the house. As the real niece arrives directly afterwards, the distracted husband is thoroughly entangled in his troubles. These are unravelled with the greatest cleverness, and at last Hummingtop is reconciled with his wife, and all ends well, if not very probably.

As Arthur Hummingtop, Mr. C. H. Hawtrey is at his very best, and his performance could hardly be improved upon. He is well supported by Mr. Glover as Ralph Omerod, his friend, whom he is "obliged to marry as well as his wife, his mother-in-law, and his brother-in-law." Miss Vane Featherstone makes a pretty Georgie, divided between love for her husband and obedience to her mother; and Miss Carlotta Zerbini is very good as the Mother-in-Law. Joshua Gillibrand is represented by Mr. W. S. Penley, and Rosa Columbier by Miss Lottie Venne; and these two, though their parts are small, contribute most unmistakeably to the success of the comedy. Together, they fairly bring down the house, especially with their song, "They are fairly in it." Mr. Penley has a most ridiculous get-up and some funny wheezes.

An "Adelphi piece" conveys to most playgoers a distinct idea of a particular kind of play, and "The Bells of Haslemere" is one of the best of this kind which has been presented at the Messrs. Gatti's theatre. It is a melodrama full of exciting incident, well worked out, and smart dialogue. Mr. William Terriss represents the young Squire of Haslemere (Frank Beresford), whose coming of age is being celebrated with great rejoicings by the whole village at the rising of the curtain. Beloved by all the villagers, he is swindled out of all his rights by Joseph Thorndyke, his trustee (Mr. Beauchamp), and a London financier, John Silkstone (Mr. Beveridge). Silkstone is, however, to be unmasked in the end by his associate, Captain Vere, an adventurer of many aliases, well played by Mr. Chas. Cartwright, and Mary Northcote, the schoolmistress (Miss Annie Irish) of the village. Of the many other characters, Miss Millward plays with much quiet dignity as the miller's sister, Evelyn Brookfield, who is engaged to the young Squire Thorndyke. The scene changes from England to America, and back to the old country again. Improbable as many of the incidents are, much virtuous sentiment, greatly applauded by the audience, is worked in, and all the tricks of the wicked scoundrels are heartily hissed by a thoroughly sympathetic audience. Messrs. Pettitt and Grundy, the authors, evidently know their audience.

### Hospitals, Doctors, Treatment, and Attendance.

### THE IN- AND OUT-PATIENTS' HOSPITAL DIARY.

This Diary is so arranged as to make some portion of it appear every week, and the whole in each monthly part. It has been very difficult to compile, and corrections will at all times be gratefully received. It has been suggested that similar information about the larger Provincial and Scotch Hospitals would be useful to many readers. We should be glad to hear if this is felt to be the case.

### GENERAL HOSPITALS—*continued.*

| Hospitals and Terms of Admission. | Physicians, Surgeons, and Medical Officers, with Days and Hours of Attendance. |
|---|---|
| **University College Hospital** (or **North London**), Gower Street, W.C. Sec., Mr. N. H. Nixon<br>Accidents and emergencies free. All other cases by Governor's letter of recommendation | *In-Physicians*, Drs W. Fox, Tu. Th. 2, S. 9.30; Ringer, M. W. F. 1.30; Bastian, M. 2.30, W. 10, F. 2 30; F. Roberts, Tu. 1.15, Th. 1.30, S 9.30<br>*In-Surgeons*, Messrs. B. Hill, Tu 2.15, W. 2, F. 2.15; C. Heath, M. W. F. 2; M. Beck, Tu. Th S. 2<br>*Out-Physicians*, Drs. Gowers, W. S.; Poore Tu. F.; Barlow, M. Th. Hour, 1.30<br>*Out-Surgeons*, Messrs. Barker, M. Th.; Goodlee, Tu. F.; Horsley, W. S. Hour, 1.30 |
| **West London Hospital**, Hammersmith Road, W. Sec. and Supt., Mr. R. J. Gilbert<br>Accidents and emergencies free at all times. Other cases require a letter of recommendation. New cases must attend at 1.30 | *In-Physicians*, Drs. G. Rogers, D. W. Hood, F. Drewitt<br>*In-Surgeons*, Messrs C. Keetley, F. Edwards, W. B. Clarke<br>*Out Physicians*, Drs. Herringham, M. Th.; Ball, Tu. F.; S. Taylor, W. S. Hour, 2<br>*Out-Surgeons*. Messrs. Ballance, Tu. F.; Weiss, M. Th.; Wainewright, W. S. Hour, 2 |
| **Westminster Hospital**, Broad Sanctuary, S.W. Sec., Mr. S. M. Quennell.<br>Accidents and urgent cases free at all times. Other cases require a letter of recommendation | *In-Physicians*, Dr. Sturges, M. Th. S.; Allchin, M. Tu F.; Donkin, Tu. W. S. Hour, 1 30<br>*In-Surgeons*, Messrs. Cowell, M. Tu. Th.; Davy, M. Tu. F.; Macnamara, M. W. S. Hour, 1.30<br>*Out Physicians*, Drs. De H. Hall, M. Th.; A. H. Bennett, Tu. F.; Murrell, W. S. Hour, 1.30<br>*Out-Surgeons*, Messrs. Cooke, M.Th.; Bond, Tu. F; Barrow, W. S. Hour, 1.30 |

### II.—SPECIAL HOSPITALS.

### CANCER.

| | |
|---|---|
| **Cancer Hospital**, Brompton, S.W. Sec., Mr. W. H. Hughes<br>Entirely free to both in- and out-patients | *In-and Out-Surgeons*, Drs. A. Marsden, W.; H. Snow, M.; F. A. Purcell, F.: Messrs. F. B. Jessett, W.; C. Stonham, Th.; C. Jennings, Tu.; W. H. Elam, S. Hour, 2 |
| London Hospital, Whitechapel Road, E. | Daily, 1.30. *See* General Hospitals |
| Middlesex Hospital, Berners Street, W. Admission free | *In-Surgeons*, Messrs. Hulke, Lawson, and Morris<br>*Out-Surgeon*, Mr. Morris, Th. 1.30 |
| **St. Saviour's Hospital**, Osnaburg Street, N.W. Sec., Mr. E. Poitiers<br>By Governors' letters | *In- and Out-Physician*, Dr. A. Kennedy, 10 30 and 4 daily |

### CHILDREN'S DISEASES.

| | |
|---|---|
| **Alexander Hospital for Children**, 18, Queen Sq., W.C. Sec., Mrs. Marsh<br>For hip disease | *Physician*, Dr. Steavenson<br>*Surgeons*, Messrs. H. Marsh, Tu. F.; A. Bowlby, M. Th. Hour, 4.30<br>(No out-patients) |
| **All Saints' Children's Hosp.**, 4, Margaret St., W.<br>For chronic joint diseases | *Surgeon*, Mr. N. Smith<br>(No out-patients) |
| **Belgrave Hosp. for Children**, 77 & 79 Gloucester St., Pimlico. Hon. Secs., Capt. Stopford, and Rev. J. Storrs<br>By letters; accidents free | *In-and Out-Physicians*, Drs. W. Hope and W. Ewart, M. Th. 9<br>*In- and Out-Surgeons*, Messrs. C. T. Dent and Margerison, Tu. F. 9 |
| **Charing Cross Hospital**, West Strand, W.C. | *Out-Physician*, Dr. Nursby, W. S. 1.30 |
| **Cheyne Hospital for Sick and Incurable Children**, 46, Cheyne Walk, Chelsea, S.W. Sec., Miss D. Evans | *Physician*, Dr. G. V. Poore<br>*Surgeons*, Messrs. T. P. Bartlett and J. Macready, attend alternate days in afternoons (No out-patients) |
| **East London Hospital for Children and Dispensary for Women**, Glamis Road, Shadwell, E. Sec., Mr. Ashton Warner.<br>By Governor's letter. Urgent cases and accidents are admitted free. | *In-Physicians*, Drs. Donkin, M. Th.; Eustace Smith, T. F.; Crocker, F.<br>*In-Surgeons*, Messrs. A. Cæsar, R. W. Parker, W. F.<br>*Out-Physicians*, Drs. Crocker, M.; Dawson Williams, Tu. Th.; Coutts, W. F. Hours, 1 and 3<br>*Out-Surgeon*, Mr. Dunn, Tu. F. 1, 3 |
| **Evelina Hospital for Sick Children**, Southwark Bridge Road, S.E. Sec, Mr. T. S. Chapman.<br>Free, but Governor's letter takesprecedence. In-patients (boys) between 2 and 10, (girls) between 2 and 12 | *In-Physicians*, Drs. F. Taylor and J. Goodhart<br>*In-Surgeons*, Messrs. H. G. Howse and R. C. Lucas<br>*Out Physicans*, Drs. N. Tirard, M. Th. 9; F. Willcocks, Tu. F. 9.<br>*Out-Surgeons*, Messrs. C. J. Symonds, S. 9; G. H. Makins, W. 9 |

### CHILDREN'S DISEASES—*continued.*

| Hospitals and Terms of Admission. | Physicians, Surgeons, and Medical Officers, with Days and Hours of Attendance. |
|---|---|
| **German Hospital**, Dalston Lane, Dalston, E. | *In-Physician*, Dr. Rasch, M. Th. 9 |
| **Great Northern Central Hosp.**, Caledonian Rd., N. | *Physicians*, Drs. Murray and Barnes, W. 2.30 |
| **Grosvenor Hospital for Women and Children**, Vincent Sq., S.W. Hon. Secs., Mr. A. J. Ram and Miss Day<br>By letter or payment. | *In- and Out Physicians*, Drs. R. Gibbons and Steavenson, Tu. W. Th. S. 2<br>*In- and Out-Surgeons*, Messrs. Smythe and Parker, Tu. W. Th. S. 2 |
| **Hospital for Sick Children**, 48 and 49, Gt Ormond St. W.C. Sec., Mr. S. Whitford<br>By Governors' letters. If without letter, the case of the applicant is investigated.<br>In-patients between 2 and 12; out-patients under 12. | *In-Physicians*, Drs. W. Cheadle, Tu. F; Sturges, W. S.; Barlow, M. Th. Hours, 9 to 11.<br>*In-Surgeons*, Messrs. H. Marsh, W. S.; E. Owen, W. S. Hours, 9 to 11<br>*Out-Physicians*, Drs. R. J. Lee and Abercrombie, M. Th.; Lubbock and Money, Tu. F. Hours, 8.30 to 10<br>*Out-Surgeons*, Messrs. Morgan and Pitts, W. S. 8.30 to 10 |
| **King's College Hospital**, Portugal Street, W.C. | *In- and Out-Physician*, Dr. T. C. Hayes, W. F. 1 |
| **Middlesex Hospital**, Berners Street W.<br>Free. Children under 3. | *Out Physician*, Dr. Duncan, W. S. 1.30 |
| **North Eastern Hospital for Children**, Hackney Road, E. Sec., Mr. A. Nixon<br>By free ticket or small payment | *In- and Out-Physicians*, Drs. W. Cayley, F. Turner, C. Semple, and W. Pasteur, M. W. Th. S. 1.30<br>*In- and Out-Surgeons*, Messrs. Waren Tay and Pollard, M. 1.30, F. 9 |
| **Paddington Green Children's Hosp.**, Paddington Green. Sec., Mr. W. H. Pearce<br>Admission free; boys under 12; girls under 14 | *In- and Out-Physicians*, Drs. T. Lauder Brunton, Ogilvie, Tu. F.; G. Laycock M. Th; S. Phillips, W. S. Hour, 9.15<br>*In- and Out-Surgeons*, Messrs. W. W. Cheyne, M. S. Boyd, F. Hour, 9.15 |
| **Royal Hospital for Children and Women**, Waterloo Bridge Road, S.E. Sec Mr. R. G. Kestin<br>Out-Patients pay 1d. on each attendance. Boys not admissible, as out-patients, over 12, or, as in-patients, over 8. | *In-Physicians*, Drs. Park, M. Th.; Gulliver, Tu. F.; Price, W.S. Hour, 2<br>*In-Surgeon*, Mr. W. H. A. Jacobson, M. Th. Hour, 2<br>*Out-Physicians*, Drs. Park, M. Th.; Dakin, W. F.; Hadden, Tu. S. Hour, 2<br>*Out-Surgeon*, Mr. E. O. Day, W. 2 |
| **St. Mary's Hospital**, Cambridge Place, Paddington | *Out-Physician*, Dr. M. Handfield Jones, M. Th. 1 30 |
| **St. Thomas's Hosp.**, Westminster Bridge Road, S.E. | *Out-Physician*, Dr. Cory, W. S. 1.30 |
| **Samaritan Free Hosp. for Women and Children**, Lower Seymour Street, W.; Branch, 1, Dorset St., W. Sec., Mr. G. Scudamore.<br>In-patients must be between 2 and 12. Out-patients Department (free) is at Edward's Mews, Duke Street, W. | *In-Physician*, Dr. W. H. Day. Daily, 2<br>*In-Surgeon*, Mr. W. A. Meredith. Daily, 2<br>*Out Physicians*, Drs. M. Prickett and Amand Routh. W. S. 1.30<br>*Out-Surgeons*, Messrs. W. A. Meredith and J. D. Malcolm, M. Th.; A. H. G. Doran and W. S. A. Griffith, Tu. F., 1.30 |
| **Victoria Hosp. for Children**, Queens-rd., Chelsea S.W. Sec., Capt. W. C. Blount, R.N.<br>Age of boys, 2 to 12 years; age of girls, 2 to 16. Admission for in-patients by subscriber's letter. Accidents and urgent cases free at all times | *In-Physicians*, Drs. J. Evans, M. Th. 2; Ridge-Jones, Tu. F. 2<br>*In-Surgeons*, Messrs. Pick, Tu. F. 9.15; Clutton, M. Th. 4.30<br>*Out-Physicians*, Drs. A. Venn, W. S. 9.30; C. Fox, M. Th. 1.30; F. D. Drewitt, Tu. F. 9; J. Philpot, M. Th. 9<br>*Out-Surgeons*, Messrs. F. Churchill, Tu. F. 10; W. Pye, M. Th. 9.30 |
| **West London Hospital**, Hammersmith Road, W. | Daily, 2. *See* General Hospitals |

### CONSUMPTION AND DISEASES OF THE CHEST.

| | |
|---|---|
| **City of London Hosp. for Diseases of the Chest**, Victoria Park, E. Sec, Mr. T. Storrar Smith<br>By subscriber's letter available for 6 weeks. | *In-Physicians*, Drs. J. Thorowgood, S.; E. Smith, M.; J. Fothergill, W.; S. West, F.; G. A. Heron, W.; V. D. Harris, Tu. Hour, 2<br>*Out-Pysicians*, Drs. V. D. Harris, Tu.; J. A. Ornerod, Th.; E. C. Beale, Tu. F., B. Fenwick, W. S.; A. Money, W. S.; L. E. Shaw, M. Th. Hour, 2 |
| **Hospital for Consumption**, Fulham rd., Brompton, S.W. Sec., Mr. H. Dobbin<br>Admission by letter of recommendation, but, where unobtainable, application may be made to the Secretary at the Hospital. | *In-Physicians*, Drs. F.S. Thompson, M.Th.; C. T. Williams, M. Th.; R. D. Powell, Tu. F.; J. Tatham, W. S.; R. E. Thompson, W. S.; F. T. Roberts, Tu. F. Hours, 2 to 4<br>*In-Surgeon*, Mr. R. J. Godlee, F.<br>*Out-Physicians*, Drs. T. H. Green, J. M. Bruce, M. Th.; J. K. Fowler, W. S.; P. Kidd and C. Y. Biss, Tu. F.; T. D. Acland, W. S. Hour, 12 |

